# South African physiotherapists’ attitudes to medicine prescription as an extension of practice

**DOI:** 10.4102/sajp.v79i1.1851

**Published:** 2023-06-23

**Authors:** Tsungirirai V. Kakono, Desmond Mathye, Sarel J. Brand, Werner Cordier

**Affiliations:** 1 Department of Pharmacology, Faculty of Health Sciences, University of Pretoria, Pretoria, South Africa; 2 Department of Physiotherapy, Faculty of Health Sciences, University of Pretoria, Pretoria, South Africa

**Keywords:** attitudes, extended scope of practice, non-medical prescribing, physiotherapy, service delivery

## Abstract

**Background:**

The extension of medicine prescription rights to other healthcare providers was proposed to reduce pharmacotherapeutic service delivery challenges in the South African healthcare sector. The scope of practice of physiotherapists is being reviewed to possibly include prescription rights to promote service delivery.

**Objectives:**

Our study assessed the attitudes of registered South African physiotherapists to the inclusion of prescription rights in their scope of practice, including enablers and challenges, and the drug classes they believe to be most relevant.

**Method:**

A cross-sectional descriptive survey of South African registered physiotherapists was completed using an online questionnaire.

**Results:**

A total of 359 participants completed the questionnaire, where 88.2% agreed that prescribing rights should be introduced, and 87.64% would want to be trained to prescribe. Participants identified several benefits: improved service delivery (91.3%); reduced healthcare delivery costs (89.8%); decreased need for multiple healthcare practitioner consultations (93.2%). Concerns included: inadequate training (55%); increased workload (18.7%); increased insurance premiums against medical liability claims (46.2%). Drugs of relevance included analgesics (95.6%) and bronchodilators (96.0%), while low preference was placed on drugs unrelated to physiotherapy. Chi-square analysis revealed associations between specific drug classes and fields of expertise.

**Conclusion:**

South African physiotherapists agree that prescribing and a limited formulary would benefit their scope of practice; however, educational concerns are evident.

**Clinical implications:**

Findings support the drive to extend the South African physiotherapy scope of practice, however, investigation will be needed to determine the most appropriate way to capacitate future physiotherapists and current graduates should the extension be approved.

## Introduction

The South African public healthcare system is strained due to a myriad of reasons, such as the quadruple burden of disease (Meyer et al. 2017), mental health concerns (Malakoane et al. 2020) and a shortage of healthcare workers, particularly in rural areas (Daviaud & Chopra 2008). Most of the South African population has access to public clinics and government hospitals. At the same time, a smaller proportion uses the private healthcare systems should they be able to afford them (Mahlathi & Dlamini 2015). Although various initiatives have been implemented to enhance healthcare service delivery and promote access, public health institutions struggle to maintain basic standards of care and patient expectations (Maphumulo & Bhengu 2019). These include the increasing patient load of healthcare professionals, the inaccessibility to appropriate healthcare (Daviaud & Chopra 2008; Neely & Ponshunmugam 2019) and increased waiting times for healthcare in rural areas (Neely & Ponshunmugam 2019). Due to prescribing regulations in South Africa, additional concerns are raised regarding obtaining prescribed medications, given the scarcity of prescribing authorities (Neely & Ponshunmugam 2019). The coronavirus disease 2019 (COVID-19) pandemic highlighted these challenges, given healthcare professional shortages and patients’ fear of seeking medical attention (Abdullahi et al. 2020). Care for COVID-19 patients was often prioritised, thus reducing medical practitioners’ availability for referrals from other healthcare professionals for prescriptions, resulting in suboptimal care and time and cost detriments (Abdullahi et al. 2020).

In South Africa, schedule 1 to 6 substances may be prescribed by authorised prescribers who are medical practitioners, dentists, veterinarians, nurse practitioners or any person registered under the *Health Professions Act, 1974*, authorised to do so (*Pharmacy Act*, 53 of 1974, 2019). As a result, medical practitioners and nurses are the only prescribing authorities generally available in primary healthcare (Daviaud & Chopra 2008). A heavily discussed way to overcome pharmacotherapeutic healthcare challenges is to broaden the scope of practice of other healthcare providers to include non-medical prescription (Noblet et al. 2018; Eales 2003); however, it should be acknowledged that healthcare provision is a multifactorial issue. Such issues include poor infrastructure (Maphumulo & Bhengu 2019), inequality between the public and private healthcare sector (Ataguba, Day & McIntyre 2015; ASSAf Standing Committee on Health 2020), inaccessibility of healthcare in many rural settings (Gaede & Versteeg 2011; Neely & Ponshunmugam 2019) and low funding expenditure for healthcare (Doherty et al. 2002; Hlafa, Sibanda & Hompashe 2019). Non-medical prescription may thus assist with affording patients more direct access to medicines, but this should be viewed within the context of the broader healthcare challenges. Physiotherapy is a dynamic profession in which changes in the scope of practice are expected and occurring already due to the modernisation of healthcare; this includes prescription rights in some countries (Unger & Lochner 2006). The United Kingdom was the first country to allow physiotherapists to prescribe medication to their patients for chronic pain and respiratory diseases (Onigbinde et al. 2013), as supplementary (dependent) prescribers from 2005 and then independent prescribers from 2013 (Chartered Society of Physiotherapy 2018).

Physiotherapists help patients develop, maintain and restore their full movement and functional ability (World Physiotherapy 2021). They can assist individuals at any stage of life where mobility and function are compromised due to age, injury, illness, conditions or environmental causes to promote their quality of life (World Physiotherapy 2021). The physiotherapy profession covers a broad and diverse range of specialities, including musculoskeletal, neurorehabilitation, cardiopulmonary, sports physiotherapy and others, which may benefit from prescribed medicines (Chartered Society of Physiotherapy 2018). In many cases, such as musculoskeletal diseases, respiratory ailments and sports injuries, physiotherapy alone may not resolve the issue, and thus adjunct pharmacotherapy may be needed (Stenner et al. 2018). Depending on the illness, pharmacotherapy may reduce pain or inflammation (analgesics and anti-inflammatory drugs), promote integumentary repair and protection (glucocorticoids), help improve functional movement (muscle relaxants) or clear the airway and improve ventilation (mucolytics and bronchodilators) (Miller 2017). Given the inability to prescribe, patients may require a referral by the physiotherapist to a prescribing authority, thus necessitating additional consultations (Miller 2017), which leads to delayed access to therapy, hindering recovery, prolonging reduced quality of life and incurring further financial burden (Stenner et al. 2018). Additional burden is also placed on prescribing practitioners, thus delaying other patients’ consultations that cannot be treated alternatively (Eales 2003).

The South African legislation currently does not allow physiotherapists to prescribe medicines; however, in 1994, the South African Society of Physiotherapists (SASP) made an effort to construct guidelines for a course to provide physiotherapists with all the knowledge required to prescribe specific drug classes (Unger & Lochner 2006). In 2004, a national survey in partnership with the Professional Board for Physiotherapy, Podiatry and Biokinetics (PPB) was developed to investigate the needs of physiotherapists regarding the administration, storage and prescription of medicines (Unger & Lochner 2006). To the best of the authors’ knowledge, there is limited published evidence available on the perception of South African physiotherapists of the inclusion of prescription rights in their scope of practice. As such, our study aimed to assess the attitudes of registered South African physiotherapists to the inclusion of prescribing into their scope of practice, their perceptions regarding benefits and concerns, and drugs that they believe would benefit the profession if prescribed by physiotherapists.

## Methods

A cross-sectional, descriptive study using an online Qualtrics questionnaire included physiotherapists registered with the Health Professions Council of South Africa (HPCSA). Given an estimated population of 7000 physiotherapists registered with the HPCSA in 2019, a sample size of 365 (as calculated using Raosoft, Raosoft Inc., Seattle, Washington, United States [US]) would allow for a 5% margin of error.

### Data collection

The questionnaire comprised of quantitative questions divided into four sections based on (1) physiotherapists’ status and experience, (2) attitudes of registered physiotherapists on prescription rights, (3) the perceived benefits and concerns of prescription rights being added to their scope of practice and (4) the drug classes that were perceived as appropriate to include in their practice. The questionnaire was piloted by distributing it to 10 registered physiotherapists, which allowed for survey optimisation prior to distribution to the larger cohort. The survey was optimised by making editorial changes and expanding on sections requiring clarification.

Permission was sought from the SASP and the Physiotherapy Association of South Africa (PASA) to distribute the questionnaire via their social media pages. Participants were recruited by distributing a recruitment letter via these platforms containing a link to the Qualtrics questionnaire. Providing consent on the first page of the questionnaire was required. However, it did not necessitate the provision of identifying information.

### Data analysis

Quantitative data were analysed using IBM^®^ SPSS^®^ Statistics 27 (IBM Corporation, Armonk, New York, US) and expressed as descriptive statistics. Statistical analysis of variance (ANOVA) and chi-square were performed after converting Likert-scale responses into numerical scales.

### Rigour

Content validity was established through an internal review among the co-authors. In contrast, face validity and internal consistency were determined through a pilot study with 10 registered physiotherapists. Internal consistency was determined to be high, with a Cronbach’s alpha of 0.777 for questions related to perceptions of prescribing rights. Feedback from the pilot study was used to reduce reasons for possible poor response rates and highlight whether enough response sections were available, whether the participants systematically missed any questions and if additional relevant options needed to be included.

### Ethical considerations

An application for full ethical approval was made to the University of Pretoria Research Ethics Committee and ethics approval was granted on 16 June 2021 (243/2021). Ethical guidelines were followed in accordance with the standards of the University of Pretoria and with the 1964 Helsinki Declaration and its later amendments.

## Results

### Demographics

A total of 359 participants completed the survey; however, as indicated later, some sections were not completed in full. Most participants were female (75.21%) and ≥ 26 years old (86.9%), possessed a bachelor’s degree as the highest qualification (79.67%) and had ≥ 10 years of experience (73.3%) (Table 1).

**TABLE 1:** Demographics of participants (*n* = 359).

Physiotherapist information	*n*	%
**Gender**		
Male	89	24.79
Female	270	75.21
Other	0	0.00
**Age**		
< 21	2	0.56
21–25	45	12.53
26–30	89	24.79
31–35	81	22.56
36–40	48	13.37
40+	94	26.18
**Highest qualification achieved**		
Bachelor (NQF 8)	286	79.67
Master (NQF 9)	59	16.43
Doctorate (NQF 10)	8	2.23
Other	6	1.67
**Years of experience**		
0–4	96	26.74
5–9	76	21.17
10–14	69	19.22
15–19	40	11.14
20+	78	21.73
**Area in which individual works**		
Urban	246	64.20
Peri-urban	69	18.00
Rural	68	17.80

NQF, National Qualifications Framework.

### Attitudes of registered physiotherapists towards the inclusion of prescribing

**FIGURE 1:**
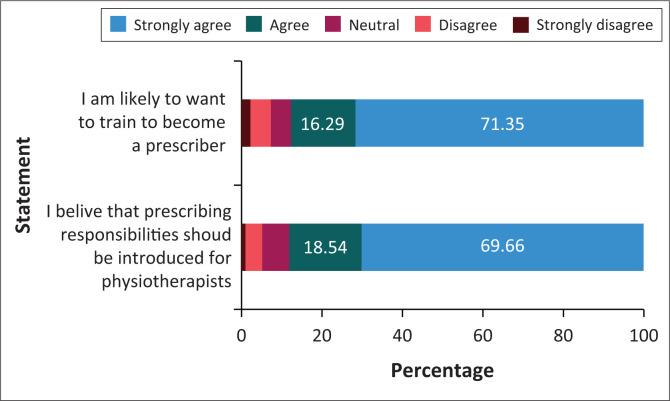
The attitudes of registered physiotherapists towards the inclusion of prescription rights into their scope of practice (*n* = 359).

**FIGURE 2:**
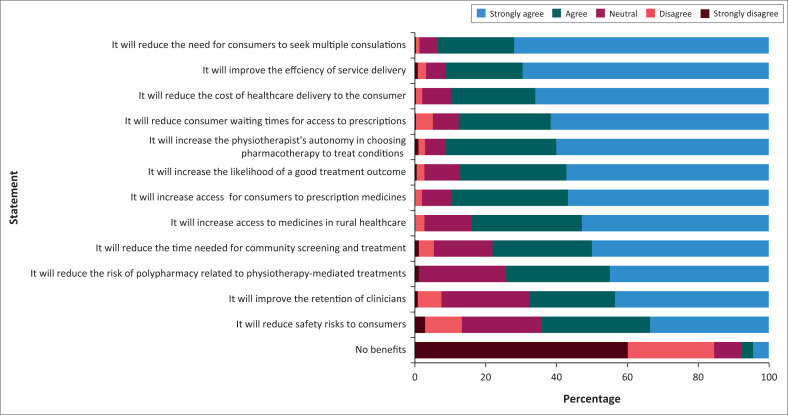
The benefits rated by the participants on the inclusion of prescription rights into their scope of practice (*n* = 359).

Most physiotherapists agreed that prescribing responsibilities should be included in their scope of practice (82.20%) and that they were willing to undergo the necessary training to comply with regulatory requirements of such an inclusion (87.64%) (Figure 1). Most participants agreed that introducing prescribing would improve the efficiency of service delivery (91.30%) and reduce the need for consumers to seek multiple consultations (93.20%), the costs of healthcare delivery to the consumer (89.8%) and consumer waiting times for access to prescriptions (87.7%) (Figure 2). A moderate number of participants agreed that prescribing would reduce safety risks to patients (64.1%) and improve the retention of clinicians (67.6%). In parallel to this, fewer than 10% of participants believed that introducing prescribing would not have any benefits.

Agreement to wanting to prescribe (as a measure from 1 [strongly agree] to 5 [strongly disagree]) was evaluated relative to age, years of experience and gender. Although the > 40-year-old group was still in high agreement with wanting to prescribe (1.84), they were statistically less likely (*p* < 0.05) to do so than participants aged 26–40 years of age (Table 2). Physiotherapists with > 20 years of experience agreed that they would want to prescribe (1.85); however, were statistically fewer (*p* < 0.05) than participants with 10–14 years of experience. No statistical difference (*p* > 0.05) was calculated between different genders. No statistical difference was observed between those working in urban, peri-urban and rural settings, although the rural cohort had the higher agreement to prescribing inclusion.

**TABLE 2:** Relationships between demographic factors and attitudes towards prescribing, where a mean closer to 1 is indicative of a greater agreement towards prescribing (*n* = 359).

Variable	Mean ± standard error of the mean
**Age**	
< 21	2.00 ± 1.00
21–25	1.56 ± 0.15
26–30	1.40 ± 0.10**
31–35	1.33 ± 0.07**
36–40	1.29 ± 0.08**
40+	1.84 ± 0.13*
**Experience**	
0–4	1.49 ± 0.10
5–9	1.34 ± 0.09**
10–14	1.38 ± 0.08**
15–19	1.42 ± 0.14
20+	1.85 ± 1.15*
**Gender**	
Male	1.19 ± 0.06
Female	1.61 ± 1.07
**Area in which individual work**	
Urban	1.56 ± 0.91
Peri-urban	1.43 ± 0.80
Rural	1.18 ± 0.49

Note: Values with

* and ** indicate a statistically significant difference between them (*p* < 0.05).

**FIGURE 3:**
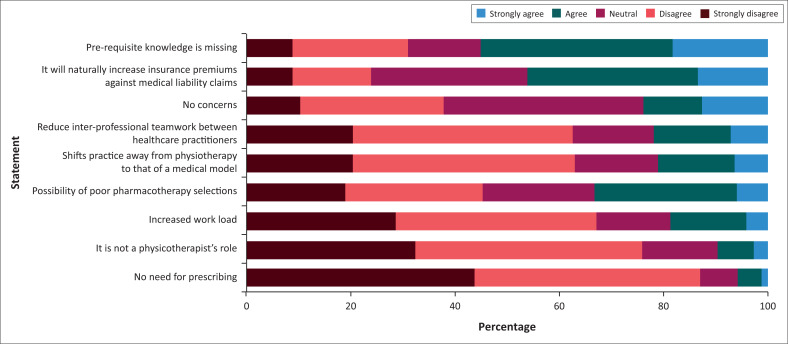
The concerns raised by the participants on the inclusion of prescription rights into their scope of practice (*n* = 359).

Key concerns highlighted by participants were that prerequisite knowledge was missing (55.00%), and insurance premiums against medical liability claims would increase (46.20%). Some also indicated concern over the possibility of poor pharmacotherapy selection (33.20%) (Figure 3). Most participants disagreed that prescribing is not a physiotherapist’s role (75.8%), would increase workload (66.8%) and that there was no need for prescribing (87%). Approximately a third (38.4%) of participants believed there were no concerns.

### Drug classes participants felt should be included in their prescribing formulary

**FIGURE 4:**
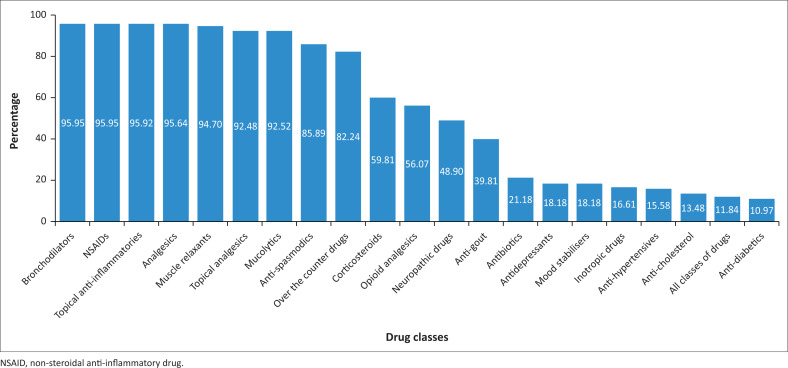
Drug classes physiotherapists believed should be included into their scope of practice (*n* = 359).

Of the list of drug classes provided to participants for rating, the following were prominently featured (> 80%): bronchodilators, non-steroidal anti-inflammatory drugs (NSAIDs), topical inflammatory agents, analgesics, muscle relaxants, topical analgesics, mucolytics, antispasmodic drugs and over-the-counter drugs (Figure 4). Antidiabetics, all classes of drugs, anticholesterol, antihypertensives, inotropic drugs, mood stabilisers, antidepressants and antibiotics were less favoured (< 25%). Other options, such as antigout, neuropathic drugs, opioids and corticosteroids, had a moderate level of agreement. Additional drug classes and/or drugs mentioned by participants included immune boosters, anabolic steroids, dermatological drugs for iontophoresis and scarring, naturopathic medicines, cannabinoids, oestrogens, botox, saline, performance-enhancing drugs, nutritional supplements, emergency drugs, homoeopathic medicines and drugs used for bladder function improvement.

Chi-square analysis between the field in which physiotherapists practised and the drugs they felt were necessary to prescribe revealed differential responses between participants (only significant findings presented in Table 3, Table 4 and Table 5, while full tables are provided in Appendix 1 Table 1-A1, Table 2-A1 and Table 3-A1).

**TABLE 3:** Relationships between field of expertise and drug classes related to pain and inflammation that were deemed significant (*n* = 359).

Field of expertise of participant	Number of individuals	Analgesics	Opioid analgesics	NSAIDs	Topical analgesics	Topical anti-inflammatories	Corticosteroids
**Amputees**							
Involved	77	96.1	71.4*	98.7	94.7	98.7	71.4
Not	208	98.6	55.8	96.6	91.8	95.7	60.6
**Chronic cardiorespiratory diseases**							
Involved	89	95.5	69.7*	94.4	88.8	92.1	67.4
Not	196	99.0	55.6	98.5	94.4	98.5**	61.7
**Chronic disease management**							
Involved	93	94.6	63.4	95.7	89.2	95.7	67.7
Not	192	99.5**	58.3	97.9	94.2	96.9	61.5
**Education**							
Involved	64	96.9	71.9*	98.4	95.3	96.9	71.9
Not	221	98.2	56.6	96.8	91.8	96.4	61.1
**Health promotion/public health**							
Involved	68	94.1	73.5**	95.6	91.2	97.1	75.0*
Not	217	99.1*	55.8	97.7	93.1	96.3	59.9
**Mental health**							
Involved	21	95.2	57.1	95.2	90.5	100.0	85.7*
Not	264	98.1	60.2	97.3	92.8	96.2	61.7
**Pain and musculoskeletal/orthopaedics**							
Involved	228	97.8	59.6	98.2*	93.0	97.4	61.4
Not	57	98.2	61.4	93.0	91.1	92.9	71.9
**Neurology**							
Involved	121	96.7	65.3	96.7	91.7	97.5	72.7**
Not	164	98.8	56.1	97.6	93.3	95.7	56.7
**Paediatrics**							
Involved	101	96.0	64.4	96.0	92.1	97.0	76.2**
Not	184	98.9	57.6	97.8	92.9	96.2	56.5
**Women’s health/continence**							
Involved	42	100.0	66.7	97.6	95.2	100.0	78.6*
Not	243	97.5	58.8	97.1	92.1	95.9	60.9

Note: Bold items with asterisks denote a statically higher desire to prescribe based on significance testing. Full version of the table is available in Appendix 1, Table 1-A1.

NSAID, non-steroidal anti-inflammatory drug.

* , *p* < 0.05;

** , *p* < 0.01.

**TABLE 4:** Relationships between field of expertise and drug classes related to muscle relaxation and pulmonary diseases that were deemed significant (*n* = 359).

Field of expertise of participant	Number of individuals	Total	Muscle relaxants	Antispasmodic	Mucolytics	Bronchodilators
Cardiorespiratory/acute medicine/surgery	Involved	126	93.7	87.2	96.0	99.2*
	Not	159	97.5	90.6	91.8	94.3
Chronic cardiorespiratory diseases	Involved	89	94.4	89.9	97.8	100.0*
	Not	196	96.4	88.7	91.8	94.9
Chronic disease management	Involved	93	97.8	92.5	96.8	100.0*
	Not	192	94.8	87.4	92.2	94.8

Note: Bold items with asterisks denote a statically higher want to prescribe based on significance testing. Full version of the table is available in Appendix 1, Table 2-A1.

* , *p* < 0.05.

**TABLE 5:** Relationships between field of expertise and drug classes associated with lifestyle diseases and others that were deemed significant (*n* = 359).

Field of expertise of participant	Number of individuals	Antibiotics	Antihypertensives	Inotropic	Antidiabetics	Antigout	Anticholesterol	Antidepressants	Mood stabilisers	Neuropathic	Over-the-counter	All classes
**Amputees**												
Involved	77	31.2	20.8	25.0	14.5	50.0	21.1	21.1	26.3	64.5*	83.1	13.0
Not	208	20.7	14.4	15.4	10.6	39.4	12.0	18.8	16.8	48.6	84.1	12.5
**Burns/plastics**												
Involved	61	31.1	23.0	25.0	15.0	46.7	25.0**	23.3	28.3*	60.0	82.0	16.4
Not	224	21.4	14.3	16.1	10.7	41.1	11.6	18.3	17.0	50.9	84.4	11.6
**Chronic cardiorespiratory diseases**												
Involved	89	33.7**	20.2	22.5	15.7	44.9	22.5**	22.5	28.1*	53.9	83.1	15.7
Not	196	18.9	14.3	15.9	9.7	41.0	10.8	17.9	15.4	52.3	84.2	11.2
**Chronic disease management**												
Involved	93	28.0	19.4	21.5	14.0	48.4	20.4*	26.9*	28.0*	62.4*	82.8	10.8
Not	192	21.4	14.6	16.2	10.5	39.3	11.5	15.7	15.2	48.2	84.4	13.5
**Education**												
Involved	64	28.1	21.9	23.4	15.6	50.0	14.4*	26.6	26.6	50.0	85.9	12.5
Not	221	22.2	14.5	16.4	10.5	40.0	11.8	17.3	17.3	53.6	83.3	12.7
**Gerontology/age decay**												
Involved	48	27.1	20.8	20.8	14.6	56.3*	20.8	20.8	27.1	58.3	91.7	10.4
Not	237	22.8	15.2	17.4	11.0	39.4	13.1	19.1	17.8	51.7	82.3	13.1
**Lymphoedema**												
Involved	25	40.0*	24.0	20.0	16.0	44.0	24.0	24.0	28.0	56.0	84.0	4.0
Not	260	21.9	15.4	17.8	11.2	42.1	13.5	18.9	18.5	52.5	83.8	13.5
**Neurology**												
Involved	121	29.8*	19.8	19.8	14.9	47.1	22.3**	24.8*	23.1	60.3*	81.8	14.0
Not	164	18.9	13.4	16.6	9.2	38.7	8.6	15.3	16.6	47.2	85.4	11.6
**Paediatrics**												
Involved	101	31.7*	20.8	23.8	15.8	48.5	24.8***	27.7**	28.7**	64.4**	87.1	14.9
Not	184	19.0	13.6	14.8	9.3	38.8	8.7	14.8	14.2	46.4	82.1	11.4
**Palliative care**												
Involved	39	33.3	23.1	25.6	15.4	51.3	23.1	23.1	28.2	69.2*	82.1	10.3
Not	246	22.0	15.0	16.7	11.0	40.8	13.1	18.8	18.0	50.2	84.1	13.0
**Rheumatology**												
Involved	35	37.1*	25.7	28.6	17.1	60.0*	25.7*	22.9	20.0	62.9	82.9	8.6
Not	250	21.6	14.8	16.5	10.8	39.8	12.9	18.9	19.3	51.4	84.0	13.2
**Rural Generalist**												
Involved	18	50.0**	33.3*	27.8	11.1	66.7*	22.2	16.7	11.1	61.1	88.9	16.7
Not	267	21.7	15.0	17.3	11.7	40.6	13.9	19.5	19.9	52.3	83.5	12.4
**Sports**												
Involved	115	20.9	15.7	17.4	12.2	42.6	13.9	14.8	14.8	43.5**	88.7	7.8
Not	170	25.3	16.5	18.3	11.2	42.0	14.8	22.5	22.5	59.2	80.6	15.9
**Women’s health**												
Involved	42	38.1*	23.8	23.8	16.7	47.6	19.0	26.2	21.4	61.9	73.8	9.5
Not	243	21.0	14.8	16.9	10.7	41.3	13.6	18.2	19.0	51.2	85.6	13.2

Note: Bold items with asterisks denote a statically higher want to prescribe based on significance testing. Full version of the table is available in Appendix 1, Table 3-A1.

* , *p* < 0.05;

** , *p* < 0.01;

*** , *p* < 0.001.

Statistically significant (*p* < 0.05) differences were observed for the following fields of expertise, indicating a greater preference for certain drugs: rheumatology (antigout, antibiotics and anticholesterol); rural generalist (antigout, antihypertensives and antibiotics); women’s health and/or continence (corticosteroids and antibiotics); amputees (neuropathic drugs and opioid analgesics); burns and/or plastics (mood stabilisers and anticholesterol); cardiorespiratory and/or acute medicine and/or surgery (bronchodilators); chronic cardiorespiratory diseases (mood stabilisers, bronchodilators, topical anti-inflammatories, antibiotics, opioid analgesics and anticholesterol) and chronic disease management (neuropathic, mood stabilisers, antidepressants, anticholesterol, bronchodilators, analgesics).

## Discussion

Several countries, such as the United Kingdom, Namibia, Brazil and the United States, have introduced non-medical prescribing rights for health professionals to alleviate the burden on their healthcare sectors (Ecker et al. 2020; Lim, North & Shaw 2017; Maier 2019). Physiotherapists practise as independent prescribers in the United Kingdom, while those in Australia and New Zealand are supplementary prescribers (Costa 2017). Non-medical prescribing has been demonstrated to improve healthcare quality and efficiency as it provides a variety of benefits that include quick access to medication, effective and safe use of medicines and improved utilisation of professional skills (Courtenay, Carey & Stenner 2011; Graham-Clarke et al. 2019; Lim, Courtenay & Fleming 2013; Teslim 2014). Our study is one of the few that has been conducted to investigate the perceptions of South African physiotherapists of the inclusion of prescription rights into their scope of practice. Participants supported including such rights and indicated an interest in training to become prescribers. Such a finding is corroborated by Unger and Lochner (2006), who also found that South African physiotherapists favour an extended scope of practice. Outside of South Africa, Nigeria, Australia, Brazil and New Zealand have also indicated a need to include a non-medical prescription for physiotherapists to improve the quality of care (Costa 2017; Morris & Grimmer 2014). Upon stratification concerning working in urban, peri-urban or rural settings, no difference in the level of agreement was observed, suggesting that the divide in accessibility was not a driving force to their opinion. However, a larger sample size will be needed to determine whether this is a true reflection thereof.

Although physiotherapists of all ages and experience were interested in prescribing, physiotherapists older than 40 or with more than 20 years of experience were less in agreement with younger and less experienced participants. Similarly, younger Nigerian physiotherapists have been reported to support the inclusion of prescription rights more than older participants (Onigbinde et al. 2013). The authors attributed this to the depth of pharmacology training, as pharmacology inclusion in physiotherapy training was a recent addition to Nigerian tertiary programmes. Similarly, Unger and Lochner (2006) reported that fewer than 54% of South African physiotherapists had pharmacology in their undergraduate training. Considering that older physiotherapists are approaching the end of their careers, they may be hesitant about the additional training needed to qualify for prescribing rights. Differences in the interest in prescribing were also noted among physiotherapists who studied at different institutions – perhaps due to subtle differences in the curricula presented at these institutions (Naidoo et al. 2018).

Our participants agreed that non-medical prescribing would improve their service delivery, benefit their patients’ recovery and reduce the potential incorrect use of medicines. Furthermore, most participants indicated the benefit to their patients. Countries like the United Kingdom have seen success in healthcare service delivery that have adopted prescription rights (Department of Health and Social Care 2005; Graham-Clarke et al. 2019). Extending the scope of practice to physiotherapists in the United Kingdom reduced patient waiting time by 76%, improved clinical management by 79% and improved patient satisfaction by 77% – 90% (ACT Health 2021). Eales (2003) reported that non-medical prescriptions would benefit patients who require prescribed medication by saving time and costs incurred by additional consultation. Allowing for non-medical prescription saves patients additional consultations, reduces the patient load on other prescribers and increases professional improvement, identity and acknowledgement (Costa 2017).

The major concern noted was the lack of pharmacological knowledge required to prescribe appropriately. Australian student physiotherapists have also emphasised the need for educational prerequisites that support prescribing competencies (Noblet et al. 2018). A study that investigated the competency of non-medical prescribers in pharmacovigilance proved that most non-medical prescribers did not feel competent enough regarding adverse drug reaction reporting (Stewart et al. 2013). This indicated a need for non-medical prescribers to have extensive training in pharmacology to guarantee that physiotherapists become well-trained prescribers (Stewart et al. 2013). Participants in our study thus understand the potential knowledge deficiency that may impact such a responsibility. Patients who depend on medications frequently consult with physiotherapists; thus, physiotherapists must understand the effects of medications, particularly adverse drug responses (Miller 2017).

Training to be a non-medical prescriber in the United Kingdom and New Zealand generally consists of full-time tertiary education, supervised practice with a designated medical practitioner (DMP) and rigorous academic and practice evaluations (Carey et al. 2020; Raghunandan, Tordoff & Smith 2017). Concerns have been raised about a potential redirection from the physiotherapy model towards a medical model, which may undermine the profession. Some participants also believed that prescribing was outside their scope of practice as physiotherapy involves the use of physical means to rehabilitate (World Physiotherapy 2021). Therefore, introducing pharmacological models may cause a deviation from the main purpose of physiotherapy. Furthermore, a significant concern raised by participants was that the inclusion would create interprofessional conflicts due to pay inequalities (Noblet et al. 2018).

Participants were asked to choose from all classes of drugs; most indicated an interest in prescribing bronchodilators, analgesics, NSAIDs, topical anti-inflammatories, muscle relaxants and mucolytics. In contrast, few indicated preferences for antidiabetics, anticholesterols, antihypertensives and inotropic drugs. Similar findings were made among Australian physiotherapists who wished to access NSAIDs (Grimmer et al. 2002) and Nigerian physiotherapists with analgesics (Teslim 2014). Analgesics, NSAIDs and muscle relaxants are important drug classes for ailments treated by physiotherapists (Teslim et al. 2014). Access to such drugs would increase patients’ response to physiotherapists’ treatment modalities, thus shortening the recovery time (Teslim 2014). Importantly, in our study, such analgesic medication did not overtly include opioids. The low agreement in including opioids by participants could be linked to the potential crisis of opioid misuse and dependency in South Africa; hence physiotherapists would instead prescribe analgesics with limited risks (Myers, Siegfried & Parry 2003).

Most participants did not support prescribing all classes of drugs, suggesting that participants believed in prescribing drugs that fall within their specific scope of practice. Similar opinions were observed in the qualitative data, where reference was made to the importance of a limited formulary appropriate for their general ailments. In doing so, it may also reduce consultation with medical practitioners for prescriptions, thus redirecting patients to consult with healthcare practitioners who are better suited to treat them and provide non-medical prescriptions (Graham-Clarke et al. 2018).

Further results showed that physiotherapists’ field of work influenced the drug classes they would be willing to prescribe. Physiotherapists working in rheumatology preferred prescribing antibiotics, antigout and anticholesterol drugs. Antibiotics have been used to treat rheumatoid arthritis (Ogrendik 2014), which may agree with their experiences in practice. Physiotherapists working with chronic cardiorespiratory diseases prefer to prescribe opioid analgesics, antibiotics, bronchodilators, anticholesterol and mood stabilisers. Our results indicate that 59.81% of the participants agreed that they would want to prescribe corticosteroids to reduce inflammation, which may promote treatment efficacy or alleviate pain (Barnes 2006).

### Strengths and limitations

Given the online nature of the survey with full accessibility, it is unknown where participants may have responded more than once or whether the distribution was as wide as expected. Furthermore, not all physiotherapists are active members of the SASP and PASA. As a result, they may not have received communication regarding this project and been able to participate in the survey. A focused review of key demographics, such as those working predominantly within rural settings or other fields of expertise, may yield more information on the unique necessity for prescribing within their context. Further investigation of the governance-related aspects of adding prescribing rights was not discussed in our study.

### Implications or recommendations

Based on our findings and active attempts by the PPB to include prescribing rights in the scope of practice of South African physiotherapists, a careful review of physiotherapy curricula will be needed to determine the optimum point of inclusion of key competency development frameworks. Such changes may include theoretical and practical assessments of pharmacotherapy skills and knowledge. At the same time, supplementary courses aimed at practising physiotherapists may contribute to the professional development of those who have already graduated. All graduates may not seek continuous professional development in prescribing, which invariably will lead to separate cohorts of professionals with extended scope of practice rights. Recommendations for future studies are to investigate the correlations between specific physiotherapy fields and specific drug classes and to highlight why most physiotherapists were hesitant with wanting to prescribe drugs such as opioids.

## Conclusion

Our study demonstrated that physiotherapists support the inclusion of prescription rights but are aware of educational shortcomings. Participants highlighted the benefits of including prescribing rights for the physiotherapy profession, patients and the healthcare sector at large. Additionally, interested parties demonstrate a clear preference for drugs that aid the treatment of ailments regularly seen in physiotherapy practice. Therefore, our results support the drive for extending the scope of practice of physiotherapists in South Africa.

## Appendix 1

**TABLE 1-A1:** Relationships between field of expertise and drug classes related to pain and inflammation.

Field of expertise of participant	Number of individuals	Analgesics	Opioid analgesics	NSAIDs	Topical analgesics	Topical anti-inflammatories	Corticosteroids
**Amputees**							
Involved	77	96.1	**71.4***	98.7	94.7	98.7	71.4
Not	208	98.6	55.8	96.6	91.8	95.7	60.6
**Burns/plastics**							
Involved	61	95.1	70.5	96.7	95.0	96.7	68.9
Not	224	98.7	57.1	97.3	92.0	96.4	62.1
**Cardiorespiratory/acute medicine/surgery**							
Involved	126	97.6	61.1	96.0	94.4	95.2	65.9
Not	159	98.1	59.1	98.1	91.2	97.5	61.6
**Chronic cardiorespiratory diseases**							
Involved	89	95.5	**69.7***	94.4	88.8	92.1	67.4
Not	196	99.0	55.6	98.5	94.4	**98.5****	61.7
**Chronic disease management**							
Involved	93	94.6	63.4	95.7	89.2	95.7	67.7
Not	192	**99.5****	58.3	97.9	94.2	96.9	61.5
**Education**							
Involved	64	96.9	**71.9***	98.4	95.3	96.9	71.9
Not	221	98.2	56.6	96.8	91.8	96.4	61.1
**Gerontology/age decay**							
Involved	48	95.8	64.6	97.9	95.8	100.0	68.8
Not	237	98.3	59.1	97.0	91.9	95.8	62.4
**Health promotion/public health**							
Involved	68	94.1	**73.5****	95.6	91.2	97.1	**75.0***
Not	217	**99.1***	55.8	97.7	93.1	96.3	59.9
**Lymphoedema**							
Involved	25	96.0	68.0	96.0	96.0	100.0	80.0
Not	260	98.1	59.2	97.3	92.3	96.1	61.9
**Mental health**							
Involved	21	95.2	57.1	95.2	90.5	100.0	**85.7***
Not	264	98.1	60.2	97.3	92.8	96.2	61.7
**Pain and musculoskeletal/orthopaedics**							
Involved	228	97.8	59.6	**98.2***	93.0	97.4	61.4
Not	57	98.2	61.4	93.0	91.1	92.9	71.9
**Neurology**							
Involved	121	96.7	65.3	96.7	91.7	97.5	**72.7****
Not	164	98.8	56.1	97.6	93.3	95.7	56.7
**Occupational health**							
Involved	24	95.8	62.5	95.8	95.8	95.8	66.7
Not	261	98.1	59.8	97.3	92.3	96.5	63.2
**Paediatrics**							
Involved	101	96.0	64.4	96.0	92.1	97.0	**76.2****
Not	184	98.9	57.6	97.8	92.9	96.2	56.5
**Palliative care**							
Involved	39	97.4	66.7	94.9	89.7	92.3	61.5
Not	246	98.0	58.9	97.6	93.1	97.1	63.8
**Rehabilitation (mixed)**							
Involved	191	97.4	60.7	96.9	92.1	96.3	65.4
Not	94	98.9	58.5	97.9	93.5	96.8	59.6
**Rheumatology**							
Involved	35	94.3	65.7	97.1	97.1	97.1	74.3
Not	250	98.4	59.2	97.2	92.0	96.4	62.0
**Rural generalist**							
Involved	18	94.4	77.8	94.4	88.9	100.0	83.3
Not	267	98.1	58.8	97.4	92.9	96.2	62.2
**Sports**							
Involved	115	98.3	55.7	98.3	93.0	97.4	61.7
Not	170	97.6	62.9	96.5	92.3	95.9	64.7
**Women’s health/continence**							
Involved	42	100.0	66.7	97.6	95.2	100.0	**78.6***
Not	243	97.5	58.8	97.1	92.1	95.9	60.9

Note: Bold items with asterisks denote a statically higher want to prescribe based on significance testing.

* , *p* < 0.05;

** , *p* < 0.01.

**TABLE 2-A1:** Relationships between field of expertise and drug classes related to muscle relaxation and pulmonary diseases.

Field of expertise of participant	Number of individuals	Total	Muscle relaxants	Antispasmodic	Mucolytics	Bronchodilators
Amputees	Involved	77	94.8	89.5	97.4	98.7
	Not	208	96.2	88.9	92.3	95.7
Burns/plastics	Involved	61	91.8	88.3	93.4	100.0
	Not	224	96.9	89.3	93.8	95.5
Cardiorespiratory/acute medicine/surgery	Involved	126	93.7	87.2	96.0	**99.2***
	Not	159	97.5	90.6	91.8	94.3
Chronic cardiorespiratory diseases	Involved	89	94.4	89.9	97.8	**100.0***
	Not	196	96.4	88.7	91.8	94.9
Chronic disease management	Involved	93	97.8	92.5	96.8	**100.0***
	Not	192	94.8	87.4	92.2	94.8
Education	Involved	64	98.4	92.2	93.8	100.0
	Not	221	95.0	88.2	93.7	95.5
Gerontology/age decay	Involved	48	100.0	91.7	95.8	95.8
	Not	237	94.9	88.6	93.2	96.6
Health promotion/public health	Involved	68	94.1	91.2	95.6	100.0
	Not	217	96.3	88.4	93.1	95.4
Lymphoedema	Involved	25	92.0	96.0	100.0	100.0
	Not	260	96.2	88.4	93.1	96.2
Mental health	Involved	21	95.2	95.2	100.0	100.0
	Not	264	95.8	88.6	93.2	96.2
Pain and musculoskeletal/orthopaedics	Involved	228	96.5	89.0	94.7	96.9
	Not	57	93.0	89.3	89.5	94.7
Neurology	Involved	121	95.0	90.1	94.2	98.3
	Not	164	96.3	88.3	93.3	95.1
Occupational health	Involved	24	100.0	83.3	100.0	100.0
	Not	261	95.4	89.6	93.1	96.2
Paediatrics	Involved	101	95.0	90.1	95.0	99.0
	Not	184	96.2	88.5	92.9	95.1
Palliative care	Involved	39	94.9	92.3	92.3	100.0
	Not	246	95.9	88.6	93.9	95.9
**Rehabilitation** (mixed)	Involved	191	96.3	89.5	93.7	97.4
	Not	94	94.7	88.2	93.6	94.7
Rheumatology	Involved	35	94.3	94.3	97.1	97.1
	Not	250	96.0	88.4	93.2	96.4
Rural generalist	Involved	18	88.9	83.3	88.9	94.4
	Not	267	96.3	89.5	94.0	96.6
Sports	Involved	115	97.4	87.0	91.3	97.4
	Not	170	94.7	90.5	95.3	95.9
Women’s health/continence	Involved	42	92.9	81.0	100.0	100.0
	Not	243	96.3	90.5	92.6	95.9

Note: Bold items with asterisks denote a statically higher want to prescribe based on significance testing.

* , *p* < 0.05.

**TABLE 3-A1:** Relationships between field of expertise and drug classes associated with lifestyle diseases and others.

Field of expertise of participant	Number of individuals	Antibiotics	Antihypertensives	Inotropic	Antidiabetics	Antigout	Anticholesterol	Antidepressants	Mood stabilisers	Neuropathic	Over the counter	All classes
**Amputees**												
Involved	77	31.2	20.8	25.0	14.5	50.0	21.1	21.1	26.3	**64.5***	83.1	13.0
Not	208	20.7	14.4	15.4	10.6	39.4	12.0	18.8	16.8	48.6	84.1	12.5
**Burns/plastics**												
Involved	61	31.1	23.0	25.0	15.0	46.7	**25.0****	23.3	**28.3***	60.0	82.0	16.4
Not	224	21.4	14.3	16.1	10.7	41.1	11.6	18.3	17.0	50.9	84.4	11.6
**Cardiorespiratory/acute medicine/surgery**												
Involved	126	25.4	16.7	18.4	13.6	44.0	17.6	20.8	23.2	56.0	86.5	14.3
Not	159	22.0	15.7	17.6	10.1	40.9	11.9	18.2	16.4	50.3	81.8	11.3
**Chronic cardiorespiratory diseases**												
Involved	89	**33.7****	20.2	22.5	15.7	44.9	**22.5****	22.5	**28.1***	53.9	83.1	15.7
Not	196	18.9	14.3	15.9	9.7	41.0	10.8	17.9	15.4	52.3	84.2	11.2
**Chronic disease management**												
Involved	93	28.0	19.4	21.5	14.0	48.4	**20.4***	**26.9***	**28.0***	**62.4***	82.8	10.8
Not	192	21.4	14.6	16.2	10.5	39.3	11.5	15.7	15.2	48.2	84.4	13.5
**Education**												
Involved	64	28.1	21.9	23.4	15.6	50.0	**14.4***	26.6	26.6	50.0	85.9	12.5
Not	221	22.2	14.5	16.4	10.5	40.0	11.8	17.3	17.3	53.6	83.3	12.7
**Gerontology/age decay**												
Involved	48	27.1	20.8	20.8	14.6	**56.3***	20.8	20.8	27.1	58.3	91.7	10.4
Not	237	22.8	15.2	17.4	11.0	39.4	13.1	19.1	17.8	51.7	82.3	13.1
**Public health promotion**												
Involved	68	29.4	22.1	23.5	11.8	45.6	14.7	25.0	22.1	57.4	80.9	13.2
Not	217	21.7	14.3	16.2	11.6	41.2	14.4	17.6	18.5	51.4	84.8	12.4
**Lymphoedema**												
Involved	25	**40.0***	24.0	20.0	16.0	44.0	24.0	24.0	28.0	56.0	84.0	4.0
Not	260	21.9	15.4	17.8	11.2	42.1	13.5	18.9	18.5	52.5	83.8	13.5
**Mental health**												
Involved	21	33.3	14.3	14.3	9.5	52.4	14.3	28.6	33.3	47.6	90.5	4.8
Not	264	22.7	16.3	18.3	11.8	41.4	14.4	18.6	18.3	53.2	83.3	13.3
**Pain musculoskeletal/orthopaedics**												
Involved	228	21.9	15.8	17.1	10.1	42.5	14.9	18.9	19.3	54.4	85.5	11.0
Not	57	29.8	17.5	21.4	17.9	41.1	12.5	21.4	19.6	46.4	77.2	19.3
**Neurology**												
Involved	121	**29.8***	19.8	19.8	14.9	47.1	**22.3****	**24.8***	23.1	**60.3***	81.8	14.0
Not	164	18.9	13.4	16.6	9.2	38.7	8.6	15.3	16.6	47.2	85.4	11.6
**Occupational health**												
Involved	24	33.3	20.8	20.8	20.8	45.8	20.8	29.2	33.3	45.8	91.7	8.3
Not	261	22.6	15.7	17.7	10.8	41.9	13.8	18.5	18.1	53.5	83.1	13.0
**Paediatrics**												
Involved	101	**31.7***	20.8	23.8	15.8	48.5	**24.8*****	**27.7****	**28.7****	**64.4****	87.1	14.9
Not	184	19.0	13.6	14.8	9.3	38.8	8.7	14.8	14.2	46.4	82.1	11.4
**Palliative care**												
Involved	39	33.3	23.1	25.6	15.4	51.3	23.1	23.1	28.2	69.2*	82.1	10.3
Not	246	22.0	15.0	16.7	11.0	40.8	13.1	18.8	18.0	50.2	84.1	13.0
**Rehabilitation (mixed)**												
Involved	191	26.2	18.8	20.4	12.0	45.0	16.8	20.4	21.5	54.5	84.8	12.0
Not	94	18.1	10.6	12.9	10.8	36.6	9.7	17.2	15.1	49.5	81.9	13.8
**Rheumatology**												
Involved	35	**37.1***	25.7	28.6	17.1	**60.0***	**25.7***	22.9	20.0	62.9	82.9	8.6
Not	250	21.6	14.8	16.5	10.8	39.8	12.9	18.9	19.3	51.4	84.0	13.2
**Rural generalist**												
Involved	18	**50.0****	**33.3***	27.8	11.1	**66.7***	22.2	16.7	11.1	61.1	88.9	16.7
Not	267	21.7	15.0	17.3	11.7	40.6	13.9	19.5	19.9	52.3	83.5	12.4
**Sports**												
Involved	115	20.9	15.7	17.4	12.2	42.6	13.9	14.8	14.8	**43.5****	88.7	7.8
Not	170	25.3	16.5	18.3	11.2	42.0	14.8	22.5	22.5	59.2	80.6	15.9
**Women’s health**												
Involved	42	**38.1***	23.8	23.8	16.7	47.6	19.0	26.2	21.4	61.9	73.8	9.5
Not	243	21.0	14.8	16.9	10.7	41.3	13.6	18.2	19.0	51.2	85.6	13.2

Note: Bold items with asterisks denote a statically higher want to prescribe based on significance testing.

* , *p* < 0.05;

** , *p* < 0.01;

*** , *p* < 0.001.
